# Genome-Wide Identification of Loci Associated With Phenology-Related Traits and Their Adaptive Variations in a Highbush Blueberry Collection

**DOI:** 10.3389/fpls.2021.793679

**Published:** 2022-01-21

**Authors:** Kyoka Nagasaka, Soichiro Nishiyama, Mao Fujikawa, Hisayo Yamane, Kenta Shirasawa, Ebrahiem Babiker, Ryutaro Tao

**Affiliations:** ^1^Graduate School of Agriculture, Kyoto University, Kyoto, Japan; ^2^Kazusa DNA Research Institute, Chiba, Japan; ^3^Thad Cochran Southern Horticultural Laboratory, United States Department of Agriculture, Agricultural Research Service, Poplarville, MS, United States

**Keywords:** *Vaccinium corymbosum*, interspecific hybridization, introgression, chilling requirement, flowering date, ripening date

## Abstract

Genetic variation in phenological traits is the key in expanding production areas of crops. Southern highbush blueberry (SHB) is a blueberry cultivar group adapted to warmer climates and has been developed by multiple interspecific hybridizations between elite northern highbush blueberry (NHB) (*Vaccinium corymbosum* L.) and low-chill *Vaccinium* species native to the southern United States. In this study, we employed a collection of diverse SHB accessions and performed a genome-wide association study (GWAS) for five phenology-related traits [chilling requirement (CR), flowering date, ripening date, fruit development period, and continuous flowering] using polyploid GWAS models. Phenology-related traits showed higher heritability and larger correlation coefficients between year replications, which resulted in the detection of robust phenotype–genotype association peaks. Notably, a single association peak for the CR was detected on Chromosome 4. Comparison of genotypes at the GWAS peaks between NHB and SHB revealed the putative introgression of low-chill and late-flowering alleles into the highbush genetic pool. Our results provide basic insights into the diversity of phenological traits in blueberry and the genetic establishment of current highbush cultivar groups.

## Introduction

In perennial crops, phenological characteristics are one of the determination factors for cultivation areas and productivity. Annually, perennial plants recur a sequence of plant developmental stages and growers are required to provide preferable growth conditions for plants to ensure stable yield and high-quality fruits (García-Tejero et al., 2010; Woznicki et al., 2015). Despite the efforts of growers, when plants were grown in areas far from their original locations, unfavorable climate alters the duration of developmental stages and possibly cause developmental defects, resulting in the reduction of yield and/or fruit quality (Muchow et al., 1990; Zheng et al., 2009). One way to overcome the geographical limitations due to the climate differences is to confer a variation in phenological characteristics in a crop gene pool, and this could be achieved by introgression using native plant species adapted to the new region.

Highbush blueberry (*Vaccinium corymbosum* L.; 2*n* = 4*x* = 48) is a tetraploid species native to North America. Highbush blueberry has been classified into northern highbush blueberry (NHB) and southern highbush blueberry (SHB) depending on their chilling requirement (CR) and winter hardiness (Retamales and Hancock, 2012). NHB typically requires 800–1,000 chilling hours (CH), whereas SHB generally requires less than 550 CH to break dormancy, induce bloom, and vegetative growth. The high CR of NHB was a major barrier to grow highbush blueberries in southern areas of the United States. The SHB breeding efforts started in 1948 and have intensified through collaboration between the United States Department of Agriculture and blueberry scientists in Arkansas, Florida, Georgia, Mississippi, and North Carolina (Moore, 1993). By utilizing high interspecific cross-compatibility, SHB cultivars have been developed by crossing NHB (*V. corymbosum*) with various native low-chill *Vaccinium* species in the *Cyanococcus* section, especially *Vaccinium darrowii* and *Vaccinium elliottii* (Lyrene, 2006). Despite the economic importance of *Vaccinium* species, the genetic basis and introgression of the adaptive traits that have been conferred during the establishment of SHB have been poorly understood, partially due to the complexity of the polyploid and species hybridization.

The growth and development of highbush blueberry are closely tied to the transition of seasons. Flower bud initiation in blueberry is associated with the short-day condition (Hall et al., 1963): in NHB cultivars, complete flower structures were seen in early November and their development ceases in early December (Gough et al., 1978). On the other hand, in the SHB cultivar “Sharpblue,” flower structures become apparent in early December and continue to develop throughout the winter (Huang et al., 1997). Highbush blueberry requires a certain period of low temperatures for uniform bud breaks; temperature between 1 and 12°C is known to be effective in breaking dormancy in NHB, among which 6°C was most effective (Norvell and Moore, 1982). After the fulfillment of CR, flower buds start to swell and finally open over a period of 3–4 weeks in the spring (Retamales and Hancock, 2012). Fruit growth in blueberry exhibits a double sigmoid curve consisting of three stages (Mainland and Eck, 1968), and the harvesting period is depending on the cultivar and the surrounding environment.

Genome-wide association study (GWAS) is a powerful tool for deciphering genetic bases underlying the diversity of phenotypes of interest. However, in polyploids, genotype calling is not as straightforward as in diploids, possibly resulting in assigning the wrong genotype to samples (Grandke et al., 2016). Even with the correct genotype, in polyploid species with polysomic inheritance, several gene action types need to be assumed, such as additive, simplex dominant, duplex dominant, and diploidized marker effect, depending on the mode of action of alleles (Rosyara et al., 2016). Despite the difficulties in the genetic characterization of polyploids with polysomic inheritance, the advent of several tools for polyploids has allowed researchers to utilize polyploid genotypes and polyploid GWAS (Bourke et al., 2018). For example, in tetraploid blueberry, which is believed to exhibit polysomic inheritance (Cappai et al., 2020), previous studies succeeded in detecting genomic regions related to fruit quality, such as fruit size and scar diameter (Ferrão et al., 2018), and volatile organic compounds (Ferrão et al., 2020). In addition to GWAS, linkage map construction and quantitative trait loci (QTL) mapping have been conducted in tetraploid blueberry populations, providing novel information on the genetic regulation of fruit firmness (Cappai et al., 2020) and fruit quality (Mengist et al., 2021).

In this study, we aimed to explore the genomic regions associated with phenology-related traits [CR, flowering date, ripening date, fruit development period (FDP), and continuous flowering] in a SHB population, which are supposed to relate to adaptation to the warmer climates. Several gene action models with polysomic inheritance were assumed to optimize GWAS analysis in polyploid species. The results led to infer geographic distribution and putative derivation of the alleles controlling adaptive traits, providing novel insights into the genetic establishment of the SHB cultivar group.

## Materials and Methods

### Plant Materials and Sequencing Data

A diverse panel of SHB genotypes and 16 NHB cultivars established at the USDA-ARS Southern Horticultural Laboratory in Poplarville, MS, United States were employed in this study (Supplementary Table 1). The panel contained released cultivars and elite breeding selections from blueberry breeding programs in Mississippi, Florida, Georgia, and North Carolina. The experimental design was a complete randomized design with four replications of each genotype. Plants were grown in a sandy loam soil (pH 5.2) amended with pine bark following recommended production practices, such as drip irrigation, summer pruning, and application of 6.5 g of 13-13-13 fertilizer per plant after harvest. Fertilizer was broadcasted on the soil surface under the plant canopy. For all the accessions, double-digest restriction site-associated DNA sequencing (ddRAD-seq) had been conducted previously (Nishiyama et al., 2021) and the data were retrieved from the database. Phenology-related traits were collected for 95 SHB genotypes from 2018 to 2020, as explained in the section “Phenotyping.”

#### Alignments

Read alignment was performed following the methods described in Nishiyama et al. (2021). Briefly, sequences with a base-quality Phred score lower than 20 and with *N* bases were trimmed, and reads shorter than 35 bp were discarded. Clean reads were mapped to the *V. corymbosum* ‘Draper’ reference genome (Colle et al., 2019) using BWA-MEM (version 0.7.17) (Li and Durbin, 2009). Among the four-phased sets of the “Draper” genome (Colle et al., 2019), we selected the longest scaffold set representing 12 ‘Draper’ homoeologous groups (Scaffolds 1, 2, 4, 6, 7, 11, 12, 13, 17, 20, 21, and 22, representing chromosomes 1–12) as representing ‘Draper’ genomic sequences to minimize the complexity. All sequences were confirmed to satisfy the following criteria: >1,000,000 mapped read counts and >0.5 mapping rate for each accession. Multi-mapped reads were filtered out, and only unique alignments were employed in this study.

#### Single Nucleotide Polymorphism Call

The SAMtools mpileup (version 1.10) (Li et al., 2009) and VarScan (version 2.3.9) (Koboldt et al., 2012) were used to create two (Variant Call Format) VCF files. One of the file included 95 SHB genotypes (hereinafter, referred to as VCFg) and was used for following GWAS analysis. The other file included 105 SHB and 16 NHB genotypes (hereinafter, referred to as VCFa), and was used for the analysis of the geographic distribution of detected GWAS peak alleles. Genotype call was made based on the FREQ field, according to the following criteria: FREQ < 5 = reference homozygous (0/0), 5 ≤ FREQ ≤ 95 = heterozygous (0/1), 95 < FREQ = alternative homozygous (1/1). The single nucleotide polymorphism (SNP) loci were filtered using VCFtools (version 0.1.17) (Danecek et al., 2011), according to the following criteria: (1) minimum depth of coverage for each individual, 20, (2) biallelic locus only, (3) maximum missing data, 0.7, and (4) minor allele frequency, 0.05. Loci that were heterozygous for all individuals were further filtered with a custom Python script. These filtering steps were performed for VCFg and VCFa, separately.

The updog package version 2.0.2 (Gerard et al., 2018) was used to call allele dosage. The depth of reference-supporting reads and variant-supporting reads were retrieved from VCFg and VCFa, and converted into input matrices for the updog package, separately. Missing sites were handled as depth = 0. Genotyping was performed using the multidog function with model = “norm” and ploidy = 4. The SNP loci were filtered using filter_snp function with the argument prop_miss < 0.05. The posterior mode of genotype (discrete reference allele dosage; hereinafter, referred to as VCFd) and the posterior mean genotype (continuous reference allele dosage; hereinafter, referred to as VCFc) were then separately formatted into matrices. As for missing sites, genotype estimates of updog were adopted because they are suggested to be more accurate than other naive approaches, such as imputation using the grand mean (Gerard, 2020).

#### Haplotype-Based Polyploid Variant Call

Haplotype-based discrete variant call was further conducted using freebayes (version 1.3.1) (Garrison and Marth, 2012). VCFg was used for generating long haplotype calls over known variants; a new VCF file was constructed with a haplotype length option of 50 (hereinafter, referred to as VCFh). The variants in VCFh were filtered according to the following criteria: (1) minimum of nine alternate allele counts in the population, (2) >65 number of samples without missing data, and (3) biallelic loci only. Loci, where all individuals have the same genotype, were further filtered with a custom Python script.

### Phenotyping

Here we have evaluated five phenology-related traits, namely, CR, flowering date (the number of days to 50% flowering, NDF), ripening date (the number of days to 50% ripening, NDR), FDP, and continuous flowering. All NDF and NDR were recorded in Julian days (number of days from January 1). All the measurements were collected from a field at the USDA-ARS Southern Horticultural Laboratory.

#### Chilling Requirement

The accumulation of CH was measured during the winters of 2018–2019 and 2019–2020 using the WatchDog weather station (Spectrum Technologies, IL, United States). Temperatures of 7°C and below were here defined as effective, and the number of hours with the effective temperature was measured in the field. For every 100 CH (100, 200, 300, 400, and 500 CH), 2–4 branches of 5–10 cm long with enough flower buds attached were collected from 2 to 4 plants of each genotype. Branches were maintained at 24°C and then rated for floral bud development after 2 weeks (in winter of 2018–2019) or 3 weeks (in winter of 2019–2020) as described by Spiers (1978). As shown in Supplementary Figure 1, each floral bud was examined for signs of bud break and given a score from stage 1 through 7. CR was expressed in CH necessary for floral buds to reach stage 4.

#### Flowering Date, Ripening Date, and Fruit Development Period

Flowering date and ripening date were evaluated for four plants of each genotype in three (2018–2020) and two (2018–2019) harvest seasons, respectively. For each year, January 1 was defined as a reference date, and NDF and NDR were counted and represented here as the number of days from the reference date. Average values were calculated for each accession and used for the following analyses. FDP was represented by ripening date minus flowering date.

#### Continuous Flowering

Here we analyzed the continuous flowering phenotype, in which the plant flowers more than once at some stage of a season in addition to the spring flowering. Continuous flowering was recorded for each genotype when first flower of a genotype was in 10% bloom from July 1 to September 30. Phenotyping was made based on the observation during multiple seasons in the field, and the genotype that showed the continuous flowering phenotype in at least one season was defined as the continuous flowering genotype. We targeted the genetic factor derived from ‘Sharpblue,’ which is a typical cultivar that shows continuous flowering phenotype in conventional cultivation under various environments. Here the genotype showing continuous flowering phenotype was designated as 1 while the others were coded as 0.

### Estimation of Broad-Sense Heritability and Genomic Heritability

Variance components for each trait were separately obtained by a model summary function of the lme4 package (Bates et al., 2015), with all sources of variation as random effect. Broad-sense heritability (*H*^2^) was calculated using the following formula:


$${\text{H}}^{2}=\frac{\delta_{g}^{2}}{\delta_{g}^{2}+\frac{\delta_{y}^{2}}{R}+\frac{\delta_{e}^{2}}{R}}$$

where δ^2^_*g*_ represents the genotypic variance, δ^2^_*y*_ represents the variance of year, δ^2^_*e*_ represents the residual variance, and R represents the number of year replications (Yamada, 2011). In addition, 90% CIs for *H*^2^ were estimated according to Knapp et al. (1985).

In addition, genomic heritability was calculated using the marker_h2 function in the R package heritability version 1.3 (Kruijer et al., 2015), with providing a relationship matrix calculated based on the VanRaden method by Gmatrix function implemented in R package AGHmatrix version 2.0.0 (Amadeu et al., 2016). Here we included year replicates in the same model by adding additional binary covariates as an indication of the year to the “covariates” argument.

### Genome-Wide Association Study

Genome-wide association study was conducted for each trait and year replication separately. In some genotypes, phenotypic data are missing in a particular year due to sample limitation, and accessions with missing phenotypic data were excluded in this analysis. All the GWAS results were visualized using the Manhattan function implemented in R package qqman (Turner, 2014). At the aim of controlling Type I error, *p* was adjusted with the *p*.adjust function in R using Bonferroni correction. The significant level was set to false discovery rate (FDR) lower than 0.1.

#### GWASpoly Package

The R package GWASpoly version 2.0 (Rosyara et al., 2016) was used to perform GWAS analysis considering gene actions specific to polyploids with polysomic inheritance. The discrete allele dosage calculated by the updog package (VCFd) and freebayes (VCFh) were applied for GWASpoly based on Q + K linear mixed model, with up to 9th principal components as population structure (Supplementary Figure 2A), and the kinship calculated by the Gmatrix function in AGHmatrix. The number of principal components was determined based on a Scree plot (Supplementary Figure 2B) and a previous study (Nishiyama et al., 2021). Five gene actions models were tested: additive, reference allele simplex dominant (simplex-dom-ref), alternative allele simplex dominant (simplex-dom-alt), reference allele duplex dominant (duplex-dom-ref), and alternative allele duplex dominant (duplex-dom-alt), where additive model considers the SNP effect proportional to the dosage of the minor allele, simplex dominant models suppose that all the heterozygotes (AAAB, AABB, ABBB) are equivalent to one of the homozygotes (AAAA or BBBB), and duplex dominant models suppose that the duplex state (AABB) has the same effect as either the simplex (AAAB) and nulliplex (AAAA) or the triplex (ABBB) and quadriplex (BBBB) states (see ref. Rosyara et al., 2016 for more detailed explanation).

#### GEMMA Software

Genome-wide association study based on a univariate linear mixed model described by Yu et al. (2006) was performed using GEMMA software (version 0.98.3) (Zhou and Stephens, 2012), which considers additive marker effect. The continuous allele dosage calculated by using the updog package (VCFc) was applied for GEMMA software. Univariate linear mixed model test for SNP-trait association was performed with kinship matrix calculated using Gmatrix function implemented in R package AGHmatrix, and population structure correction based on up to 9th principal components. The kinship matrix was prepared as described in section “GWASpoly Package.”

#### Putative Derivation of Loci Associated With the Phenological Traits

To identify the derivation of the candidate alleles detected by GWAS analysis, we employed the population structure analysis results conducted by Nishiyama et al. (2021). The group proportion data with the optimal K value 9 was retrieved. Genotype differences at the highest peak among individuals were tested for each subpopulation.

### Statistical Analysis

Spearman’s correlation analysis was used to test the significance of the relationship (*p* < 0.05) of phenotypic variation between traits and year replications. Kolmogorov–Smirnov test and Bartlett’s test were first conducted to test the normality of distribution and equal variance, respectively. Tukey–Kramer test along with ANOVA or non-parametric Steel–Dwass test was then used to test the significance of differences (*p* < 0.05) of the phenotypic values among the genotypes at GWAS peak or original locations. For the loci with no reference homozygous or alternative homozygous states in the population (e.g., reference homozygous and heterozygous, but no alternative homozygous), *t*-test or the non-parametric Wilcoxon rank-sum test was applied (*p* < 0.05). The Chi-square test was used to examine the differences in the ratio of individuals that showed continuous flowering between original locations.

### Estimation of Linkage Disequilibrium

Linkage disequilibrium extent was estimated following the methods based on the quantile regression, as described in Nishiyama et al. (2021). Briefly, *r*^2^ values for the correlation between each SNP and all other SNPs on a chromosome were calculated based on the continuous genotype. The *r*^2^ values were regressed against physical distance for each SNP, based on quantile regression and smoothing with a cubic spline using the qsreg function implemented in the R package fields (version 9.8.6) (Nychka et al., 2017).

## Results

### Variation of the Phenology-Related Traits

**FIGURE 1:**
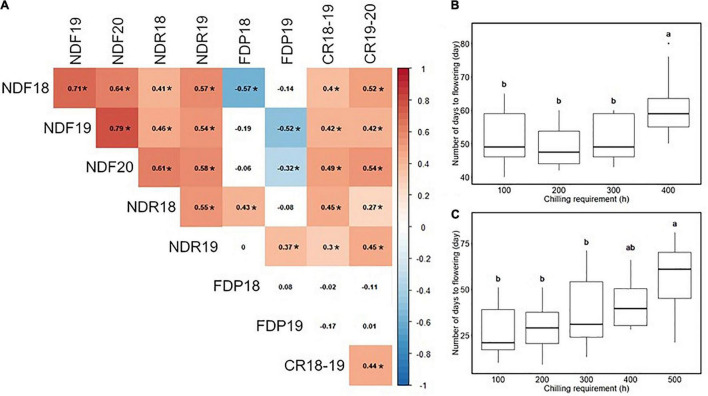
Correlation analysis among the phenology-related traits. **(A)** The result of Spearman’s rank correlation analysis. Values in cells represent Spearman’s correlation coefficient. The two-digit values at the phenotype ID represent the experimental year. Significant correlations (*p* < 0.05) were highlighted by color and indicated by asterisk (*). **(B)** Relationship between chilling requirement (CR) in 2018–2019 season and the number of days to flowering in 2019. **(C)** Relationship between CR in 2019–2020 season and the number of days to flowering in 2020. Different letters represent significant differences (*p* < 0.05).

Most traits exhibited similar average values in multiple experiment years, except that the flowering date in 2020 was 15 days earlier than in 2019 (Table 1 and Supplementary Figure 3). As for the continuous flowering, 23 SHB accessions matched our criteria for the continuous flowering (Supplementary Figure 3J). Spearman’s correlation analysis revealed the significant correlation between year replicates except for FDP (Figure 1A). In our population, flowering date, ripening date, and CR were positively correlated with each other. It was shown that the accessions with larger CR required a longer time for floral bud development and dormancy release, and bloomed later in the season (Figures 1B,C).

**TABLE 1:** Phenology-related traits of southern highbush blueberry (SHB) collection.

Phenotype	Phenotype	No. of days to flowering (day)	No. of days to ripening (day)	Development period (day)	Chilling requirement (h)
2018	mean	59 (Feb. 28)	132 (May 12)	72	NR
	SD	10.4	6.4	10.1	NR
2019	mean	53 (Feb. 22)	132 (May 12)	79	265
	SD	8.4	7.3	7.6	114
2020	mean	38 (Feb. 7)	NR	NR	285
	SD	17.9	NR	NR	148

**FIGURE 2:**
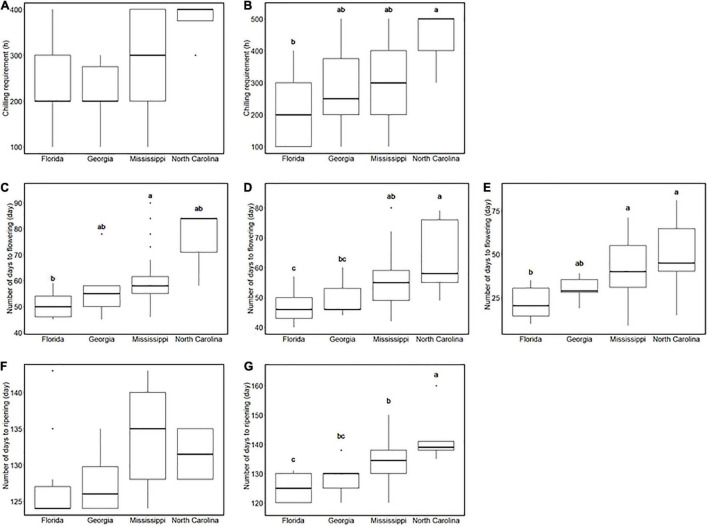
Comparison of phenology-related traits between breeding locations. **(A,B)** Chilling requirement of southern highbush blueberries (SHBs) in **(A)** the 2018–2019 season and **(B)** the 2019–2020 season for each breeding location. **(C–E)** Flowering date of SHBs in **(C)** 2018, **(D)** 2019, and **(E)** 2020 seasons for each breeding location. **(F,G)** Ripening date of SHBs in **(F)** 2018 and **(G)** 2019 seasons for each breeding location. Different letters represent significant differences (*p* < 0.05).

Southern highbush blueberry in this study are derived from four breeding locations in the United States: from south to north, Florida, Georgia, Mississippi, and North Carolina (Supplementary Figure 3K). In our environment, SHB from the northernmost breeding location required the longest CR (Figures 2A,B). Similarly, the breeding location is associated with flowering date: the accessions from the southern locations flowered earlier than those from the northern locations (Figures 2C–E), and their fruits also ripened earlier (Figures 2F,G). On the other hand, FDP and the continuous flowering did not show significant differences between breeding locations (Supplementary Figure 4).

**FIGURE 3:**
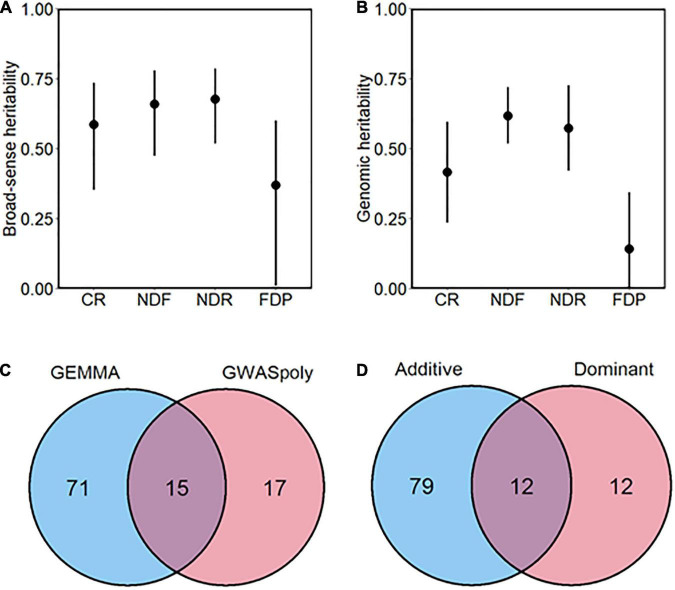
Overview of the genome-wide association study (GWAS) analysis conducted in this study. **(A)** Broad-sense and **(B)** genomic heritability of each phenology-related trait. Bars represent 90% CI. **(C)** The total number of significant phenotype-genotype associations detected by two GWAS methods. **(D)** The total number of significant phenotype-genotype associations detected by two gene action models.

In addition, we assessed the broad-sense heritability, except for the discrete continuous flowering trait (Figure 3A). Heritability of CR, NDF, NDR was moderate to high (*H*^2^ > 0.5), whereas that of FDP was relatively low, which is consistent with the low correlation coefficient between year replications. Genomic heritability, which refers to the proportion of variance explained by genome-wide markers (de Los Campos et al., 2015), was slightly lower but showed similar trend as *H*^2^ (Figure 3B).

### Comparison of the GWAS Models

A total of 65,145, 33,659, and 15,095 variants were detected with VarScan (VCFg), the updog package (VCFc and VCFd), and freebayes (VCFh), respectively. These variants were tested for phenotype-genotype association with two GWAS methods: GWASpoly and GEMMA.

Genome-wide association study analyses detected a total of 114 variant loci significantly associated with five phenology-related traits (FDR < 0.1). According to the number of associated variants, GEMMA showed the larger detection power than GWASpoly (Figure 3C). Among the significant associations, 85.4% (88 out of 103 loci) were detected by either of the GWAS models.

We categorized the GWAS models into two groups based on gene actions: Additive and Dominant. The Additive group includes additive models in GWASpoly and GEMMA software, which assume polygenic additive marker effect. The Dominant group includes simplex dominant and duplex dominant models in GWASpoly, which assume dominant gene actions. Among two groups, GWAS models belonging to the Additive group detected the largest number of variants significantly associated with traits (Figure 3D). However, the Additive group appeared not comprehensive because variant loci detected only by the Dominant group existed.

### Chilling Requirement

**FIGURE 4:**
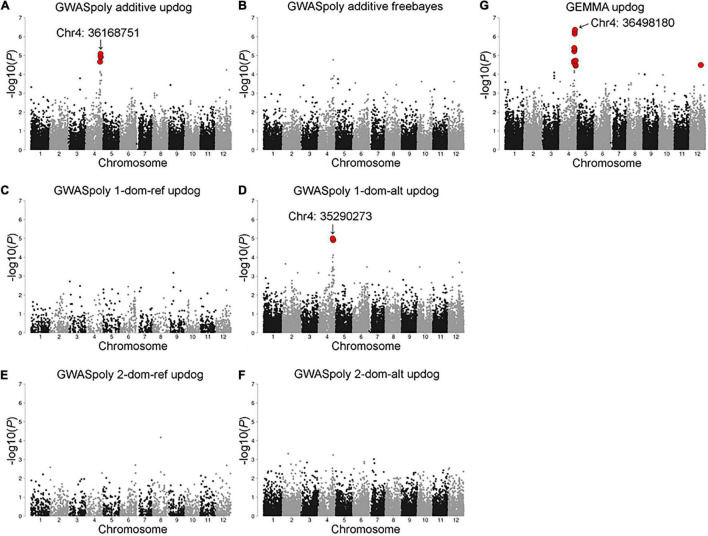
Model selection for the CR in the 2018–2019 season. Genome-wide association study result by **(A)** GWASpoly additive model with single nucleotide polymorphism (SNP) discrete genotype call, **(B)** GWASpoly additive model with haplotype-based genotype call, **(C)** GWASpoly reference simplex dominant model, **(D)** GWASpoly alternative simplex dominant model, **(E)** GWASpoly reference duplex dominant model, and **(F)** GWASpoly alternative duplex dominant model, **(G)** GEMMA with continuous genotype call. The red dots represent significant phenotype-genotype associations [false discovery rate (FDR) < 0.1].

**FIGURE 5:**
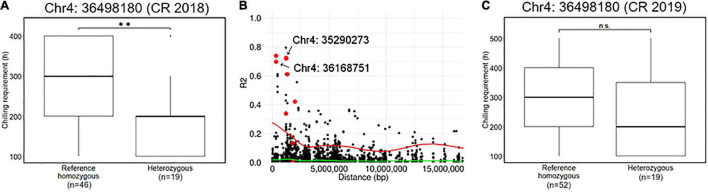
Characterization of a locus significantly associated with the CR. **(A)** Comparison of CR in 2018–2019 season based on the genotype at the significant locus, Chr4: 36498180. **(B)** Pairwise allelic genotype correlation plot of Chr4: 36498180 with all other loci on chromosome 4. Red plots represent the other loci significantly associated with CR in the 2018–2019 season detected by GEMMA software (i.e., Figure 4g). Red and green lines represent a cubic spline fitted for the 95th and 50th percentiles, respectively. **(C)** Comparison of CR in 2019–2020 season based on the genotype at the significant locus, Chr4: 36498180. Double asterisk (**) represents the significant difference (*p* < 0.01) and n.s. is an abbreviation for non-significant.

Association analyses for CR in winter of 2018–2019 allowed the identification of 12 variants located on chromosomes 4 and 12 (Figure 4 and Supplementary Table 2). The variant with the lowest *p* (Chr4: 36498180) was detected by GEMMA software with additional 10 significant variants distributed between 34,516,343 and 38,282,633 bp on chromosome 4 (Figure 4G). The comparison of CR in winter of 2018–2019 revealed that CR of the individuals with reference homozygous genotype at the top variant locus was longer than those with heterozygous genotype (Figure 5A). In addition, GWASpoly package detected significant phenotype-genotype associations at the same region as GEMMA (Figures 4A,D), though the most significant variant locus detected by an additive model in GWASpoly (Chr4: 36168751) and that by the alternative simplex dominant model (Chr4: 35290273) did not correspond to the most significant variant locus by GEMMA software. Consequently, the linkage disequilibrium (LD) pattern around the peak region was analyzed. LD analysis revealed that the significant variant loci from three different association studies were in a strong LD relationship (*r*^2^ > 0.6) with each other (Figure 5B). Thus, all three association studies detected the same LD block associated with CR. Additionally, GEMMA software detected some other significantly associated variants located in the LD block (Figure 5B). As for the other GWAS models, a similar association peak on chromosome 4 was observed, however, it was not above the genome-wide significant level (Figure 4B). With the data for the winter of 2019–2020, a significant marker effect at Chr4: 36498180 was not observed as in the 2018–2019 season (Figure 5C). In the 2019–2020 season, on the contrary to winter of 2018–2019, there were no significant peaks detected by any of the models, whereas the variant with a lower *p* by additive model in GWASpoly using haplotype-based genotype (Chr4: 36768530) was observed in the same region with that in winter of 2018–2019 (Supplementary Figure 5B). Unexpectedly, CR was significantly higher for the heterozygous accessions at Chr4: 36768530 (Supplementary Figure 5H), which implied the complexity of the CR regulation by the peak region on chromosome 4.

### Flowering Date

**FIGURE 6:**
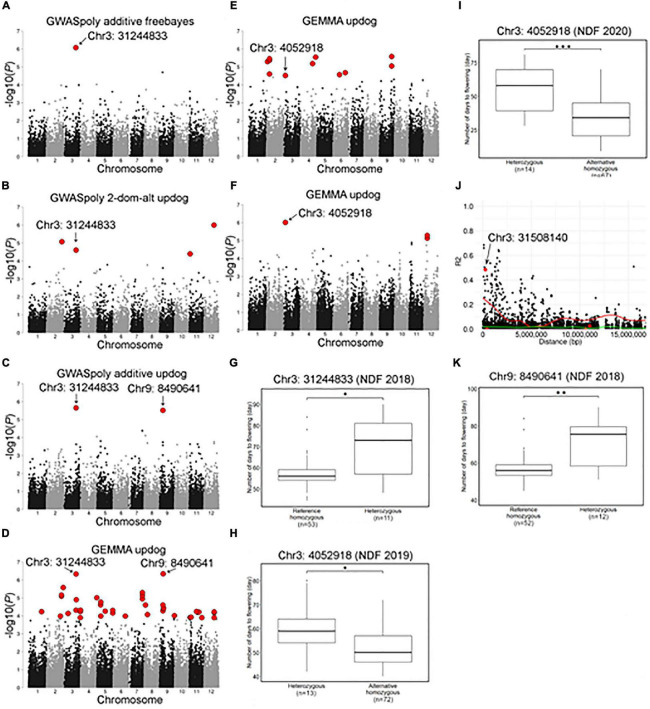
Characterization of a locus significantly associated with flowering date on chromosome 3 and 9. **(A–F)** Genome-wide association study results for the flowering date. Red dots represent significant phenotype-genotype associations (FDR < 0.1). **(A–D)** Genome-wide association study for the flowering date in 2018 detected by **(A)** GWASpoly additive model with haplotype-based genotype call, **(B)** GWASpoly alternative duplex dominant model with SNP discrete genotype call, **(C)** GWASpoly additive model with SNP discrete genotype call, and **(D)** GEMMA with continuous genotype call. **(E)** GWAS for the flowering date in 2019 detected by GEMMA with continuous genotype call. **(F)** GWAS for the flowering date in 2020 detected by GEMMA with continuous genotype call. **(G)** Comparison of flowering date in 2018 based on the genotypes at Chr3: 31244833. **(H,I)** Comparison of flowering date in **(H)** 2019 and **(I)** 2020 seasons based on the genotypes at Chr3: 4052918. **(J)** Pairwise allelic genotype correlation plot of Chr3: 31244833. Red dot indicates significant loci detected by GEMMA. Red and green lines represent a cubic spline fitted for the 95th and 50th percentiles, respectively. **(K)** Comparison of flowering date in 2018 based on genotypes at Chr9: 8490641. Asterisks (*, **, and ***) represents significant difference (*p* < 0.05, *p* < 0.01, and *p* < 0.001, respectively).

Association studies with flowering dates detected a total of 67 significant variant loci distributed across 12 chromosome groups (Figure 6 and Supplementary Table 2). Among the association peaks, those on chromosomes 3, 7, and 9 were detected by association studies in multiple years or by multiple GWAS models (Figures 6A–F and Supplementary Figures 6A,B). The significant variants on chromosome 3 were detected for 3 years of the experiment, and two loci, Chr3: 31244833 and Chr3: 4052918, underlie the significance (Figures 6A–F). Comparison of NDF by different genotypes at the significant loci revealed that SHB accessions with homozygous genotype bloomed earlier than those with heterozygous genotype (Figures 6G–I), whereas those two loci were located apart and not associated with each other. A significant locus detected on chromosome 3 by GEMMA software, Chr3: 31508140, was strongly associated with the top locus, Chr3: 31244833 (Figure 6J).

The significant variant on chromosome 9, Chr9:8490641, was detected only by association studies for NDF in 2018 (Figures 6C,D). Interestingly, similarly to the above significant variants, SHB accessions with homozygous genotype at this locus bloomed earlier (Figure 6K).

The significant variant on chromosome 7, Chr7: 10849491, was detected by association studies for NDF in 2019 and 2020 (Supplementary Figures 6A,B). As opposed to the above significant loci, SHB accessions with either nulliplex (AAAA), simplex (AAAB), or duplex (AABB) genotype bloomed earlier than those with either triplex (ABBB) or quadriplex (BBBB) (Supplementary Figures 6C,D). In the analysis for the 2018 data, a significant variant was found at 10 bp apart (Chr7: 10849505) (Supplementary Table 2), which showed similar marker effect as Chr7: 10849491 (Supplementary Figure 6E) and was strongly associated with Chr7: 10849491 (Supplementary Figure 6F).

### Ripening Date

**FIGURE 7:**
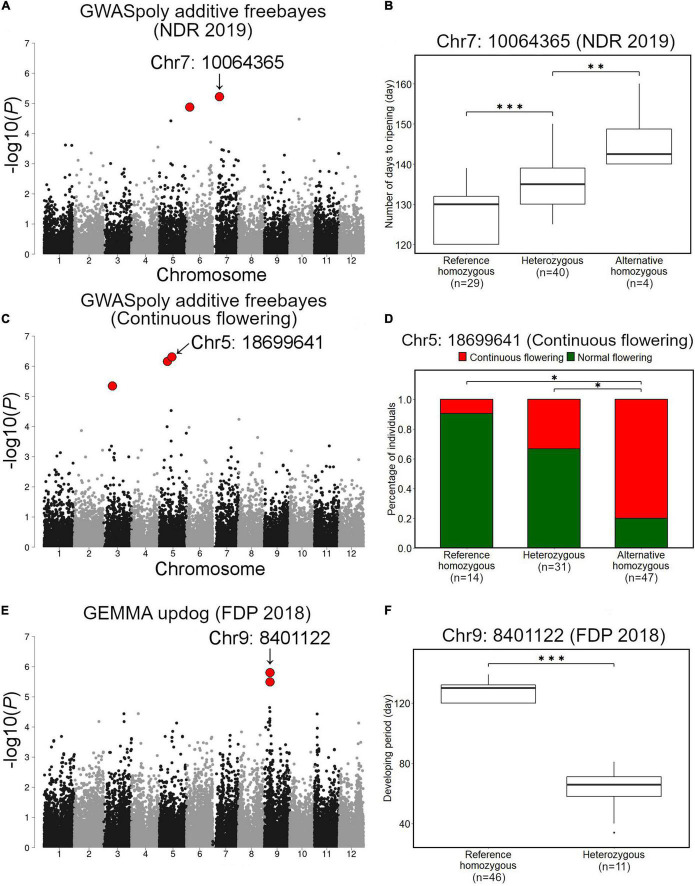
Association studies for ripening date, continuous flowering, and fruit development period (FDP). **(A,B)** Associations with ripening date. **(A)** GWAS result for ripening date in 2019 by GWASpoly additive model with haplotype-based genotype call. **(B)** Comparison of ripening date in 2019 based on the genotypes at the significant locus, Chr7: 10064365. **(C,D)** Associations with continuous flowering. **(C)** GWAS result for continuous flowering by GWASpoly additive model with haplotype-based genotype call. **(D)** Comparison of percentage of accessions with continuous flowering habit based on the genotypes at Chr5: 18699641. **(E,F)** Association with fruit development period. **(E)** GWAS result for fruit development period by GEMMA with continuous genotype call. **(F)** Comparison of fruit development period based on the genotypes at Chr9: 8401122. Red dots in **(A,C,E)** represent significant phenotype-genotype associations (FDR < 0.1). Asterisks (* and ***) in **(B,D,F)** represent significant difference (*p* < 0.05 and *p* < 0.001, respectively).

Association studies with NDR identified 18 variant loci located on chromosomes 1, 2, 4, 5, 6, 7, 9, 10, and 11 (Supplementary Table 2). Among these, we focused on a significant variant on chromosome 7 (Chr7: 10064365), which was highly significant and showed notable allele frequency pattern (Figure 7A). Comparison of NDR suggested that an allele at this locus additively regulates NDR, and fruit from SHB with reference homozygous genotype at the locus ripened earlier (Figure 7B). We could not detect any significant phenotype-genotype association for NDR in 2018.

### Continuous Flowering

Association studies with the continuous flowering detected 3 significant variant loci located on chromosomes 3 and 5 (Figure 7C and Supplementary Table 2). Among these, a variant locus detected on chromosome 5 (Chr5: 18699641) was the most significant. Comparison of the ratio of canonical flowering (single flowering in a year) SHB and continuous flowering SHB revealed that the gene action for Chr5: 18699641 is additive (Figure 7D).

### Fruit Development Period

Fruit development period was the most unstable trait in this study. Association studies detected 3 variant loci located on chromosomes 9 and 10, whereas all of them were exclusively detected by a single GWAS model (Figure 7E and Supplementary Figure 7 and Supplementary Table 2). Two variants on chromosome 9 (Chr9: 8401122 and Chr9: 8401111) were found to be significantly associated with FDP in 2018 (Figure 7E). Genotypes of SHB individuals at Chr9: 8401122 and Chr9: 8401111 were highly correlated (data not shown). In this locus, the FDP of SHB individuals with reference homozygous genotype was significantly longer than that of heterozygous ones (Figure 7F).

### Geographic View of Genotype Fractions of the Significant Loci

Here we investigated the changes in allele frequencies at the loci mentioned above across the SHB breeding locations. Most of the significant variants were supposed to be robust in our population because they are supported by multiple-year replications and/or GWAS models. In other cases, representative variants were selected based on the value of *p*.

**FIGURE 8:**
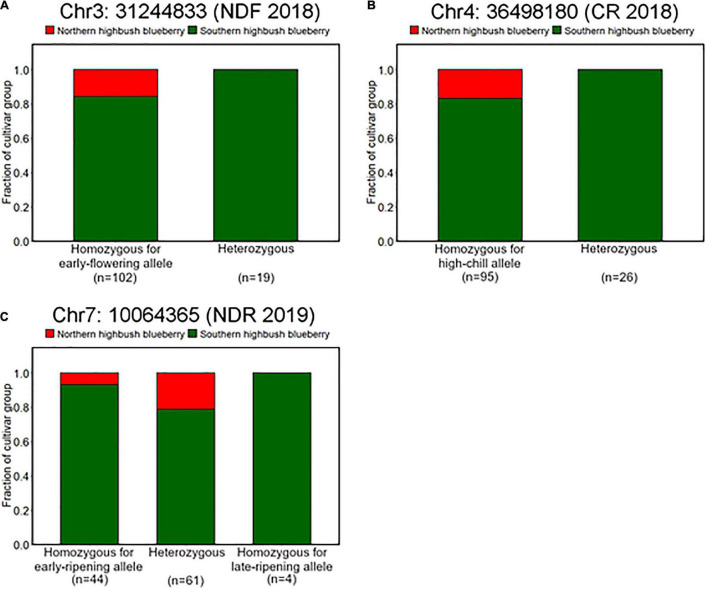
Difference of allele frequencies at loci significantly associated with phenology traits between northern highbush blueberries (NHBs) and SHBs. Proportions of cultivar groups at loci significantly associated with **(A)** flowering date in 2018 (Chr3: 31244833), **(B)** CR in 2018–2019 season (Chr4: 36498180), and **(C)** ripening date in 2019 (Chr7: 10064365).

It was observed that genotype fraction at Chr4: 36498180, associated with CR (Figures 4, 5), significantly differs between SHB and NHB, where all the tested NHB have the homozygous genotype for high-chill allele at this locus (Figure 8B). In addition, we found that all the NHB in our population had a homozygous genotype for early-flowering allele at loci significantly associated with NDF (Figure 8A). Whereas there was no NHB homozygous for the late-ripening allele at the significant peak (Figure 8C). Collectively, we assumed that genotypes of NHB at all these loci have biases toward homozygous genotype. As for the other reliable variant loci, no tendency like this was observed (Supplementary Figure 8).

**FIGURE 9:**
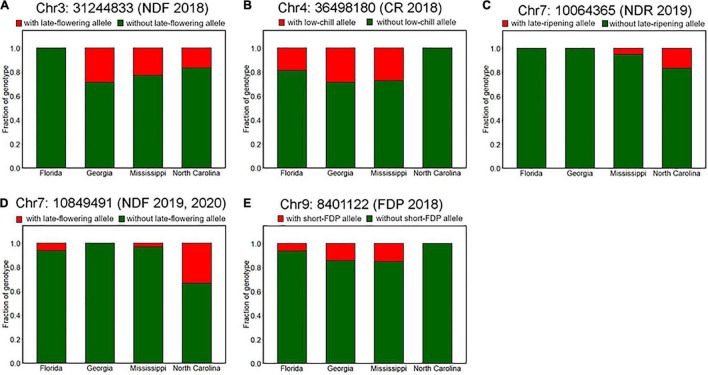
Difference of allele frequencies at the phenology-associated loci among breeding locations. Proportions of genotypes at loci significantly associated with **(A)** flowering date in 2018 (Chr3: 31244833), **(B)** CR in 2018–2019 season (Chr4: 36498180), **(C)** ripening date in 2019 (Chr7: 10064365), **(D)** flowering date in 2019 and 2020 (Chr7: 10849491), and **(E)** FDP in 2018 (Chr9: 8401122).

We further characterized the differences of genotype fractions between the breeding locations. Among all the robust variant loci detected by GWAS analysis, only the fraction of genotypes at Chr7: 10849491, which is associated with NDF (Supplementary Figure 6), was significantly different between breeding locations (Figure 9D). According to the assumed duplex dominant effect of this locus (Supplementary Figures 6C,D), SHB accessions were classified into two groups: group1 (AAAA, AAAB, and AABB) and group2 (ABBB and BBBB), where A and B represent early-flowering allele and late-flowering allele, respectively. It was shown that the fraction of group2 in SHB cultivars bred in North Carolina was significantly larger than that in Georgia and Mississippi (Figure 9D). Moreover, no SHB accessions that have group2 genotype were bred in Georgia.

Although there was no significance, some other genotypes also exhibited possible trends of allelic fixation or introgression (Figure 9). For example, SHB from the southernmost breeding location Florida had only homozygous genotype at loci significantly associated with NDF (Figure 9A), whereas this was the case for SHB from the northernmost breeding location North Carolina, which had only homozygous genotype at loci significantly associated with CR and FDP (Figures 9B,E). Besides, at the loci associated with NDR, late-ripening alleles were only found for the SHB from the northern locations (North Carolina and Mississippi) (Figure 9C). These results were largely consistent with the phenological characteristics that may be preferable in each breeding location. As for the other reliable variant loci, trends similar as above were not observed (Supplementary Figure 9).

## Discussion

### Genetic Variation of Phenology-Related Traits in the Blueberry Collection

#### Correlation Between Phenology-Related Traits

In this study, significant correlations in most pairs of the phenology-related traits in a SHB population were observed (Figure 1A). A positive correlation between flowering date and ripening date was previously reported. Campa and Ferreira (2018) investigated six phenological traits in the cultivated blueberry species (*V. corymbosum*, *Vaccinium virgatum*, *Vaccinium macrocarpon*, and *Vaccinium uliginosum*), and found that values of the correlation coefficient for trait pairs were positively or negatively significant in most cases. In addition, Rowland et al. (2020) reported a positive correlation between CR and flowering date in a diploid pseudo-backcross population (*V. darrowii* × *V. corymbosum*). Our results supported this association is conserved in a tetraploid cultivated population. The phenological traits analyzed in this study are fundamental factors to determine the suitable production area for blueberries; higher CR is advantageous to avoid early spring frost damage. In fact, this correlation has been reported in several fruit species (Ruiz et al., 2007, 2018; Fan et al., 2010; Gao et al., 2012). It is known that not only low temperatures during endodormancy but also high temperatures during ecodormancy are critical factors for determining blooming time. For example, in Japanese apricot (*Prunus mume*), Kitamura et al. (2017) suggested that flowering date was affected more by the heat requirement during ecodormancy than the CR of endodormancy. The middle-level correlation coefficient between CR and flowering date may be attributed to this, and further investigation into genetic bases underlying the heat requirement of blueberries may be meaningful for expanding the blueberry production area in the future.

#### Effect of Heritability on the Detectability of Phenotype–Genotype Associations

In this study, the phenology-related traits exhibited moderate to high broad-sense heritability (*H*^2^ > 0.5) except FDP (Figure 3A), indicating that most phenological traits are highly genotype-dependent. Although the broad-sense heritability of CR in this study (*H*^2^ = 0.59) is smaller than that in a previous study (*H*^2^ = 0.86) (Rowland et al., 2014), we detected the robust peak significantly associated with CR (Figure 4). The results supported the effectiveness of the phenotype-genotype association study for the phenological traits measured in our SHB population.

#### Comparison of GWAS Methods

In this study, GWAS analysis with GEMMA software using continuous reference allele dosage identified larger number of phenotype-genotype associations (Figure 3C), generating an ideal tower-like peak in some cases (Figure 4G). The univariate linear mixed model implemented in GEMMA software in combination with continuous genotype has already been applied to SHB and succeeded in detecting candidate loci for blueberry flavor (Ferrão et al., 2020). Here, significant SNPs detected with GEMMA software encompassed all the SNPs detected with the additive models implemented in the GWASpoly package, implying the superiority of continuous genotype call in our dataset. It has been repeatedly highlighted that discrete genotype calling may introduce errors in the data, resulting in the loss of information, and in polyploid species, it becomes increasingly difficult to assign exact discrete genotype classes, especially in higher ploidy and with sequencing data at lower read depth (Tumino et al., 2016; Yamamoto et al., 2020; Liao et al., 2021). Grandke et al. (2016) conducted an association study using regression methods and concluded that continuous genotype was superior to discrete genotypes. Besides, the superiority of continuous genotype was reported in the context of genomic selection in tetraploid blueberry (de Bem Oliveira et al., 2019). Our result is consistent with this idea and empirically supports the advantage of continuous genotype call.

In addition, some differences in the results of GWAS using discrete genotype call were observed depending on the genotyping method. In particular, GWAS with haplotype-base genotype call showed significant peaks for NDR and continuous flowering, which was not obtained by the other models (Figure 7). This may be due to a better effect of haplotype-based analysis on sequence diversity in polyploid species rather than focusing on a single SNP.

As for the marker effect assumed in association studies, additive models identified a larger number of variant-trait associations (Figure 3D). This result is consistent with the previous research in a tetraploid potato diversity panel, reporting that the additive model detected the largest number of QTLs (Rosyara et al., 2016). However, it is noteworthy that some variant loci were not detected by the additive model, while they were detected by the dominant model (Figure 3D). This limitation of detectability has already been confirmed by Rosyara et al. (2016). They showed that the statistical power of the GWAS model is higher when the assumed gene action in the GWAS model matches the gene action at unobserved QTL. Since the effect of each marker distributed across the whole genome on phenotype cannot be confirmed before performing GWAS analysis, it is worth trying several GWAS models to avoid missing significant phenotype-genotype associations. In fact, Ferrão et al. (2018) reported that GWAS models assuming non-additive gene models identified significant SNP-trait associations in a SHB population, while the conventional additive models failed to detect them. In addition, especially when population size and/or SNP density is small, in which the statistical power for detection is expected to be small, non-additive models may play an important role to find the gene model matched to the unobserved QTL (Klein, 2007; Spencer et al., 2009). In blueberry breeding and research community, since different types of tetraploid blueberry are adapted to only certain climates, it is challenging to have access to a large diversity panel in a single location. In addition, given the fact that blueberry was domesticated from few founders with intensive interspecific hybridizations, we can assume that the dominant model fits the situation to some extent, and produces favorable effects. Collectively, the use of both additive and dominant models might optimize the outcome of the association studies for phenological traits in our relatively small population size.

### Key Trait for Differentiation of NHB and SHB

Chilling requirement is one of the major differences between NHB and SHB (Retamales and Hancock, 2012), and thus its genetic control mechanism encompasses the clues to decipher the genomic characteristics of SHB. Here we detected a single robust peak on chromosome 4, whose allele frequency was in accordance with environmental differences among breeding locations (Figures 4, 9B). It should be noted that we here analyzed the phenotype of an SHB population so that the association is assumed to underlie the diversity of CR among SHB individuals, and thus the low-chill allele was found in only a part of the SHB individuals. This genotype pattern agrees with the previously proposed polygenic adaptation of SHB to the southern climates (Nishiyama et al., 2021).

Nishiyama et al. (2021) detected SNP loci (Chr1: 25303016, Chr2: 26391780, and Chr8: 23005240) that are associated with a component partially discriminating SHB from NHB and located within Mb-scale haplotype blocks. However, none of the variant loci associated with the phenology-related traits in this study were located on the genomic regions nearby the above SNP loci. Since the differences between NHB and SHB are not limited to CR, we assumed that the SNP loci detected by Nishiyama et al. (2021) were associated with traits other than CR, such as cold/heat hardiness.

Qi et al. (2021) conducted linkage map construction and QTL analysis for CR in a diploid pseudo-backcross interspecific mapping population [(*V. darrowii* × *V. corymbosum*) × *V. corymbosum*] and found one major QTL for CR detected on chromosome 5. Our GWAS peak for CR did not correspond to the result from Qi et al. (2021). However, interestingly, significant variant related to continuous flowering was detected on chromosome 5. Generally, highbush blueberry blooms once per year, whereas some SHB cultivars shows continuous flowering. This phenotype is known to be influenced by the climate of the production area (Hummer et al., 2007; Pescie et al., 2011), and thus, even though there were no significant difference in CR between normal and continuous flowering accessions in our collection (data not shown), they could be related to each other at the genetic level. Currently, it is unclear whether the QTL and GWAS peaks are identical, and further studies are required for the association between CR and continuous flowering.

### Possible Introgression and Allele Fixation During SHB Breeding History

**FIGURE 10:**
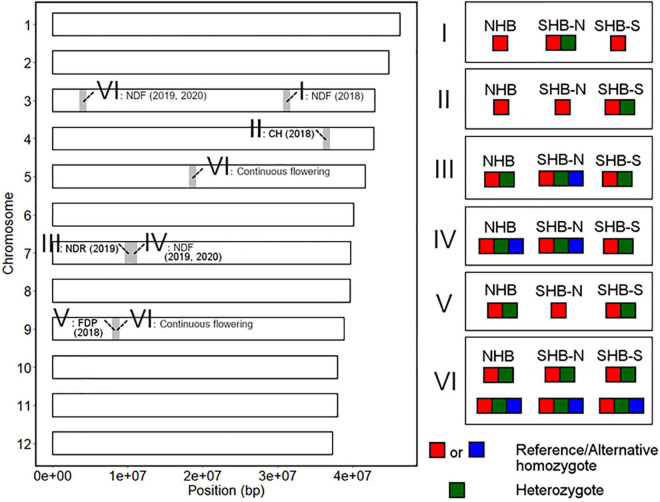
Summary of the genotype patterns of the phenology-associated loci in the highbush population. The left figure represents the chromosomal position of the detected loci. The significant loci were classified according to the genotype pattern in the highbush population as shown in the right figure. SHB-N and SHB-S represent SHB from southern and northern breeding locations, respectively.

We here summarized the robust loci detected by GWAS analysis and the differences in allele frequencies at these loci between NHB, SHB from southern breeding locations (SHB-S), and SHB from northern breeding locations (SHB-N). In this section, putative introgression and allele fixation were discussed by categorizing genotype patterns shared within each gene pool (Figure 10).

First, we hypothesized the introgression of the low-chill allele into the highbush gene pool (Figure 10 II). Here, an alternative allele at Chr4: 36498180 was designated as a low-chill allele according to the genotype pattern at the locus (Figure 5A). In this locus, all the NHBs were homozygous for the high-chill allele, while SHB was either homozygous for the high-chill allele or heterozygous genotype (Figure 8B). Besides, since SHB from North Carolina did not have the alternative allele (Figure 9B), it is indicated that the introgression of the low-chill allele has occurred predominantly in southern breeding locations, which is consistent with the result showing that SHB from the northernmost breeding location (North Carolina) had the largest CR (Figures 2A,B).

Second, allele frequency changes were observed at the variants significantly associated with NDF (Figure 10 I, IV, and VI). As for the variants at Chr3: 31244833, the late-flowering allele was suggested to be introduced into NHB (Figure 10 I). Here, an alternative allele at Chr3: 31244833 was designated as late-flowering alleles, based on the marker effect at these loci (Figure 6G). As with the CR, none of the NHB have the late-flowering alleles at these loci (Figure 8A). In the same way as NHB, SHB accession from the southernmost breeding locations (Florida) did not have the late-flowering alleles (Figure 9A). The acquisition of the late-flowering alleles might contribute to the late-flowering characteristic of SHB from northern breeding locations (Figures 2C–E). In fact, late-flowering SHB was developed to offer better protection from spring frosts (Draper, 1997), which was consistent with the observed introgression pattern. As for the variants on chromosome 7 significantly associated with NDF, both NHB and SHB possess all three possible genotype states (Supplementary Figure 8C), while the fraction of genotype states was significantly different between SHB breeding locations (Figure 9D). Possession of the late-flowering alleles on chromosome 7 was specific to SHB-N, and this was in agreement with that on chromosomes 3 (Figure 10 I). As for the other variants associated with NDF mentioned above (Chr3: 4052918 and Chr9: 8490641), no such trends of allele frequencies were observed between NHB, SHB-S, and SHB-N (Supplementary Figures 8A,F, 9A,C).

Third, the alternative allele at Chr7: 10064365 was designated as a late-ripening allele based on the marker effect (Figure 7B). Although none of the NHB in this study had a homozygous genotype for the late-ripening allele, the allele frequency was not different between NHB and SHB (Figure 8C). Since only SHB from Mississippi or North Carolina possessed homozygous genotype for late-ripening allele (Figure 9C), the increase of late-ripening allele assumed to be specific to SHB-N (Figure 10 III), whereas further investigation with a larger population, especially with NHB, needs to be conducted for understanding the changes in ripening-related QTL.

Finally, we designated an alternative allele at Chr9: 8401122 as the short-FDP allele (Figure 7F). Both NHB and SHB possessed two genotype states (Supplementary Figure 8E), while SHB from North Carolina did not have the short-FDP allele (Figures 9E, 10 V). Basically, longer FDP is not a preferable trait for the blueberry market. Our results highlighted the presence of QTL controlling FDP that may have the potential for further improving the commercial value of blueberry.

### Derivation of Alleles Introgressed to the Highbush Gene Pool

As discussed above, we found putative introgression of alleles that are supposed to be advantageous in the highbush blueberry industry (Figure 10). Here we further discuss the putative derivation of alleles introduced to the highbush gene pool by employing the result of structure analysis conducted by Nishiyama et al. (2021). Interestingly, the late-ripening allele (Figure 10 III) was suggested to be derived from subpopulation 1 predominating in rabbiteye blueberry germplasm (Supplementary Figure 10C), which was represented by blue color in Nishiyama et al. (2021). It is known that the contribution of rabbiteye germplasm to the SHB gene pool was important for improving their heat tolerance, whereas the harvest season of rabbiteye blueberries is generally later than that of highbush blueberries. Although future studies would be needed, there may be room for further improvement of SHB regarding the ripening date based on the current SHB diversity. As for the late-flowering allele and low-chill allele (Figure 10 I and II), it was suggested that they are derived from subpopulations 6 and 4 (represented by light green color and yellow color in Nishiyama et al. (2021)), respectively (Supplementary Figures 10A,B). Since the *Vaccinium* species that represent subpopulations 4 and 6 is unknown, further population structure analysis including *Vaccinium* species, such as *V. darrowii* and *V. elliottii*, which substantially contributed to the SHB population (Lyrene, 2006), is needed to elucidate the exact derivation of each phenological trait from the genetic viewpoint.

## Conclusion

In this study, genetic regions significantly associated with phenology-related traits in the SHB population were characterized based on GWAS analysis. Phenology-related traits were significantly correlated with each other in most cases, and moderate to high heritability of phenology-related traits facilitated detection of robust variant-trait associations. By comparing the genotype fraction at robust GWAS peaks between NHB and SHB, putative allele introgressions and sweeps during SHB breeding were traced and discussed. Further population structure analysis may allow unraveling the derivation of alleles contributing to the diversity of phenology-related traits in SHB.

## Data Availability Statement

The original sequence data presented in the study can be found in the DDBJ Sequence Read Archive (accession number DRA009951).

## Author Contributions

SN, HY, EB, and RT conceived and designed the study. EB and MF collected the phenotype data. KN, SN, and KS developed the bioinformatics approach. KN and SN conducted the data analysis. KN, SN, and EB drafted the manuscript. All authors interpreted the data and approved the manuscript.

## Conflict of Interest

The authors declare that the research was conducted in the absence of any commercial or financial relationships that could be construed as a potential conflict of interest.

## Publisher’s Note

All claims expressed in this article are solely those of the authors and do not necessarily represent those of their affiliated organizations, or those of the publisher, the editors and the reviewers. Any product that may be evaluated in this article, or claim that may be made by its manufacturer, is not guaranteed or endorsed by the publisher.

## Supplementary Material

Supplementary Figure 1 Observation method for the chilling requirement. Pictures show the appearance of the flower buds at each stage.

Supplementary Figure 2 Principal component analysis of genotypes based on 95 southern highbush blueberry (SHB) accessions used in this study. **(A)** Population structure. **(B)** Scree plot.

Supplementary Figure 3 Overview of the phenotype data distribution. **(A,B)** Chilling requirement (CR) in the **(A)** 2018–2019 and **(B)** 2019–2020 seasons. **(C–E)** Number of days to flowering in **(C)** 2018, **(D)** 2019, and **(E)** 2020. **(F,G)** Number of days to ripening in **(F)** 2018 and **(G)** 2019. **(H,I)** Fruit development period (FDP) in **(H)** 2018 and **(I)** 2019. **(J)** Continuous flowering. **(K)** Breeding location.

Supplementary Figure 4 Comparison of phenology-related traits between breeding locations. **(A,B)** Fruit development period in the **(A)** 2018 and **(B)** 2019 seasons. **(C)** Percentage of accessions with continuous flowering habit.

Supplementary Figure 5 Genome-wide association study (GWAS) for CR in the 2019–2020 season. GWAS results detected by **(A)** GWASpoly additive model with single nucleotide polymorphism (SNP) discrete genotype call, **(B)** GWASpoly additive model with haplotype-based genotype call, **(C)** GWASpoly reference simplex dominant model with SNP discrete genotype call, **(D)** GWASpoly alternative simplex dominant model with SNP discrete genotype call, **(E)** GWASpoly reference duplex dominant model with SNP discrete genotype call, and **(F)** GWASpoly alternative duplex dominant model with SNP discrete genotype call, **(G)** GEMMA based on continuous genotype call. **(H)** Comparison of CR in 2019–2020 season based on the genotype at Chr4: 36768530. Arrow shows the loci with the lowest value of *p*. Double asterisk (**) represents the significant difference (*p* < 0.01).

Supplementary Figure 6 Characterization of the associations with flowering date detected on chromosome 7. **(A,B)** Associations with the flowering date. **(A)** GWAS for the flowering date in **(A)** 2019 and **(B)** 2020 detected by GWASpoly reference duplex dominant model with SNP discrete genotype call. Red dots represent significant phenotype-genotype associations (FDR < 0.1). **(C,D)** Comparison of flowering date in **(C)** 2019 and **(D)** 2020 based on the genotypes at Chr7: 10849491. **(E)** Comparison of flowering date in 2018 based on the genotypes at Chr7: 10849505. Asterisks (*, **, and ***) represents the significant difference (*p* < 0.05, *p* < 0.01, *p* < 0.001), respectively. **(F)** Pairwise allelic genotype correlation plot of Chr7: 10849491. Red and glean dots represent loci significantly associated with the flowering date in 2019 and 2020, and with flowering date in 2018, respectively. Red and green lines represent a cubic spline fitted for the 95th and 50th percentiles, respectively.

Supplementary Figure 7 Manhattan plot for FDP in 2019 season. GWAS results detected by GWASpoly reference duplex dominant model with SNP discrete genotype call. The red dot represents significant phenotype-genotype associations (FDR < 0.1).

Supplementary Figure 8 Differences of allele frequencies at loci significantly associated with phenology traits between NHB and SHB. Proportions of cultivar group at loci significantly associated with **(A)** flowering date in 2019 and 2020 (Chr3: 4052918), **(B)** continuous flowering (Chr5: 18699641), **(C)** flowering date in 2019 and 2020 (Chr7: 10849491), **(D)** FDP in 2018 (Chr9: 8401122), and **(E)** flowering date in 2018 (Chr9: 8490641).

Supplementary Figure 9 Differences of phenology-related allele frequencies among breeding locations. The proportion of genotype at loci significantly associated with **(A)** flowering date in 2019 and 2020 (Chr3: 4052918), **(B)** continuous flowering (Chr5: 18699641), and **(C)** flowering date in 2018 (Chr9: 8490641).

Supplementary Figure 10 Comparison of the putative ancestral genome proportions based on the genotypes at the GWAS peaks. **(A)** Comparison of fraction of the subpopulation 6 with or without late-flowering allele at Chr3: 31244833. **(B)** Comparison of fraction of the subpopulation 4 with or without low-chill allele at Chr4: 36498180. **(C)** Comparison of fraction of the subpopulation 1 with or without late-ripening allele at Chr7: 10064365. Asterisks (*, **, and ***) represents the significant difference (*p* < 0.05, *p* < 0.01, *p* < 0.001), respectively.

Supplementary Table 1 List of accessions used in this study. Circles in the genome-wide association study (GWAS) column represented accessions employed in GWAS.
