# Supplementary material for: Genome-Wide Identification of Loci Associated With Phenology-Related Traits and Their Adaptive Variations in a Highbush Blueberry Collection
Source: Front Plant Sci. 2022 Jan 21;12:793679. doi: 10.3389/fpls.2021.793679 (PMC8814416; doi:10.3389/fpls.2021.793679)
Supplement: Supplementary Figure 1 — Observation method for the chilling requirement. Pictures show the appearance of the flower buds at each stage. [file Presentation_1.pptx]

## Slide 1
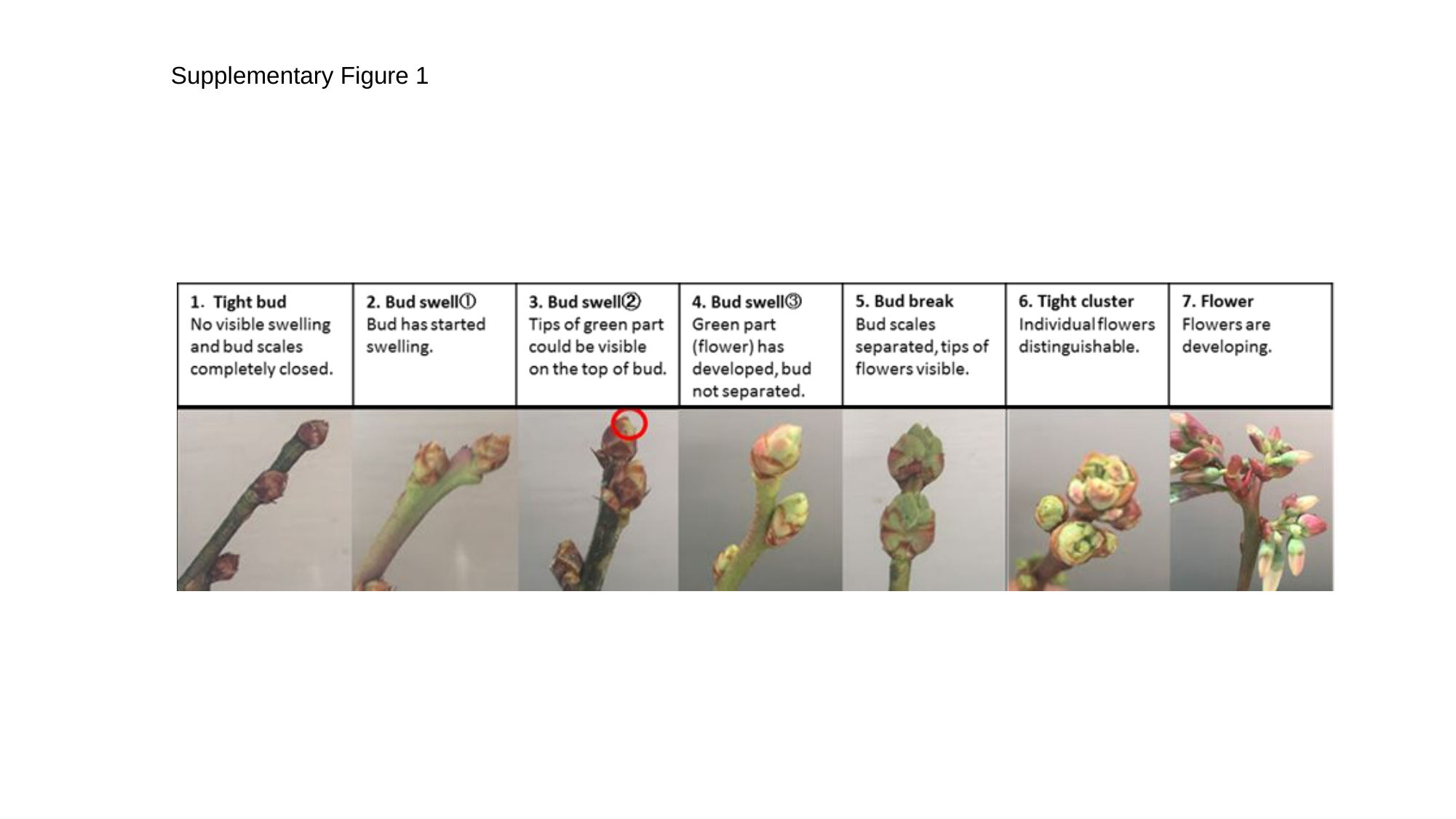

Supplementary Figure 1

## Slide 2
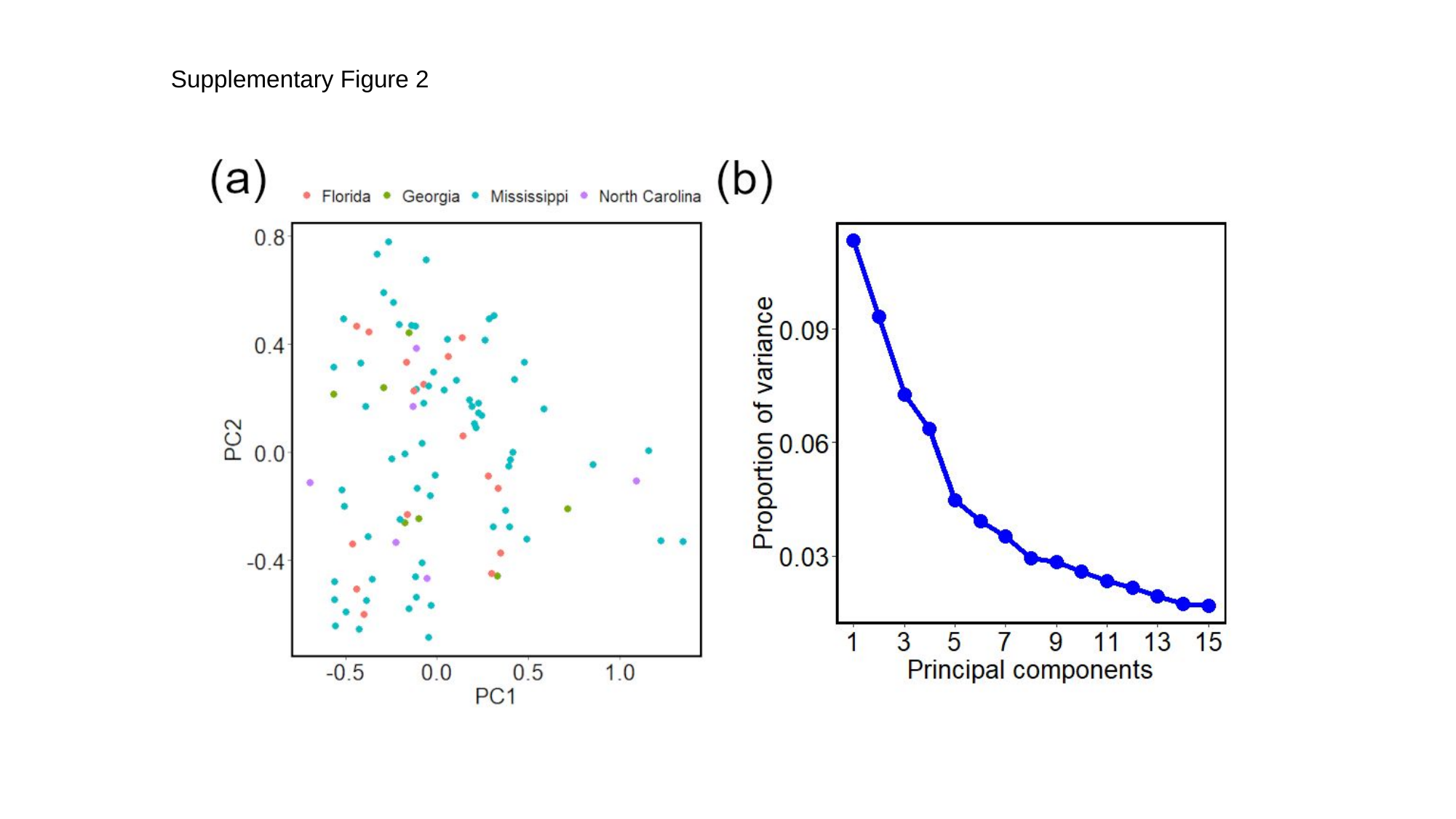

Supplementary Figure 2

## Slide 3
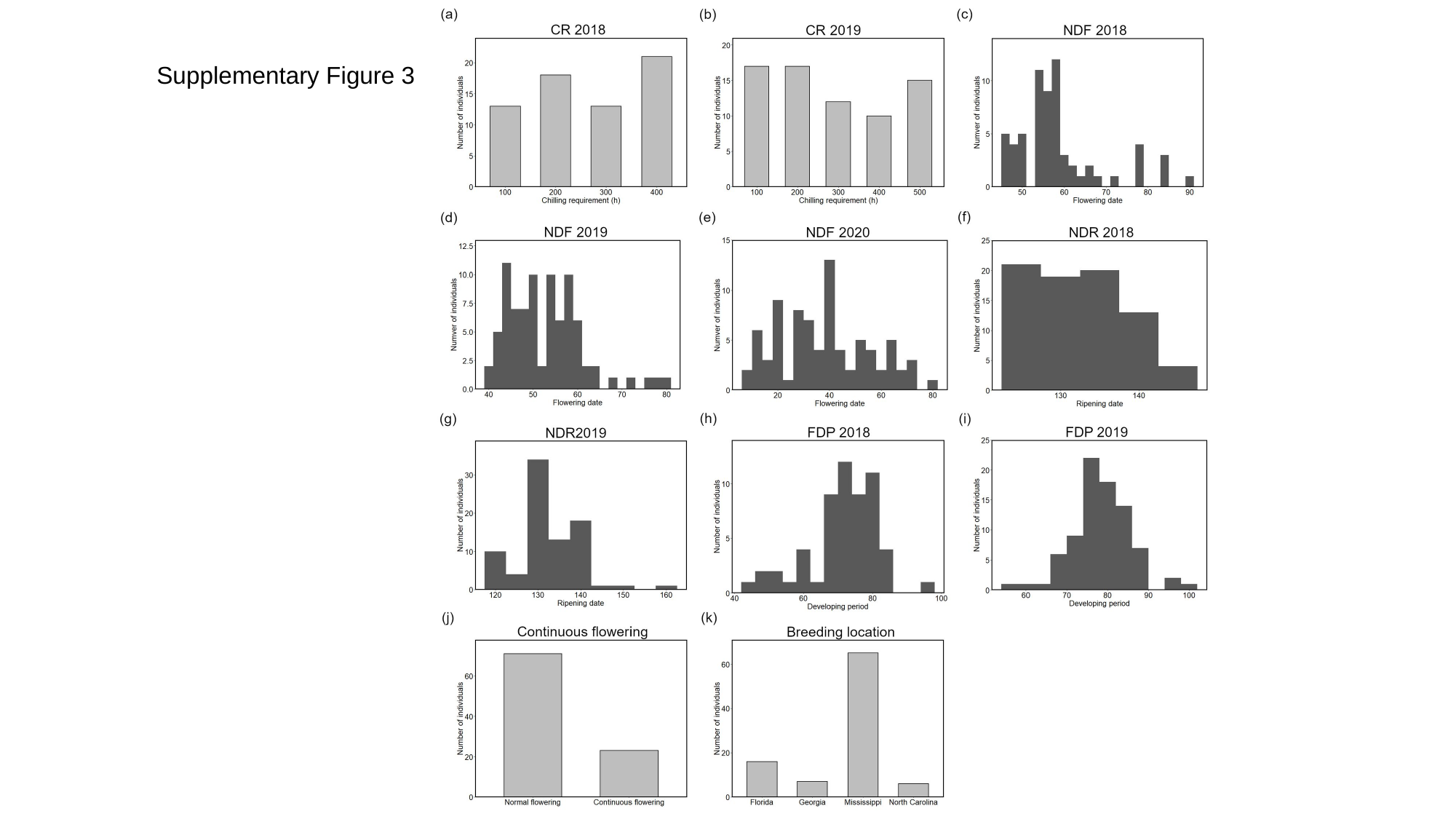

Supplementary Figure 3

## Slide 4
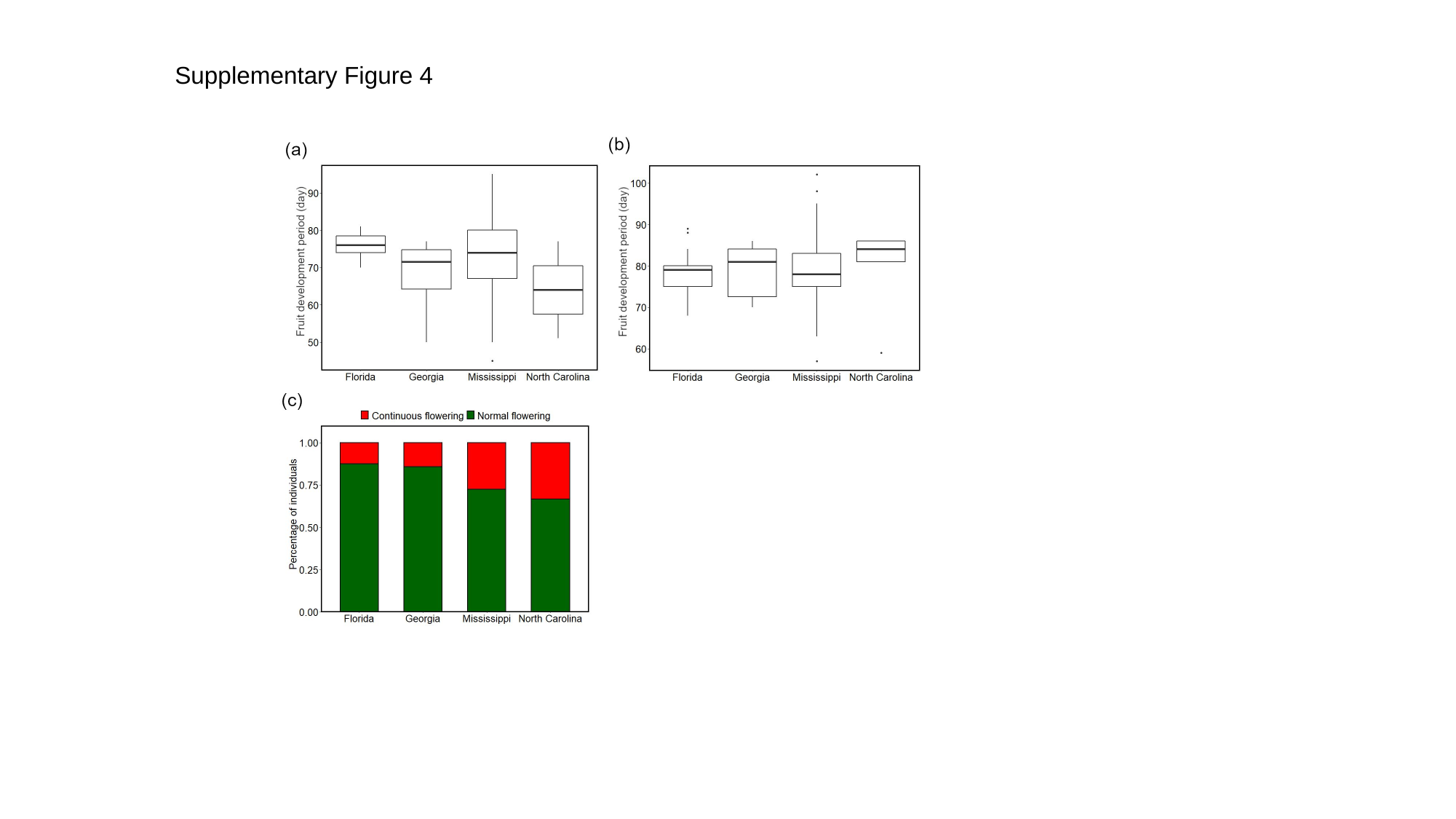

Supplementary Figure 4

## Slide 5
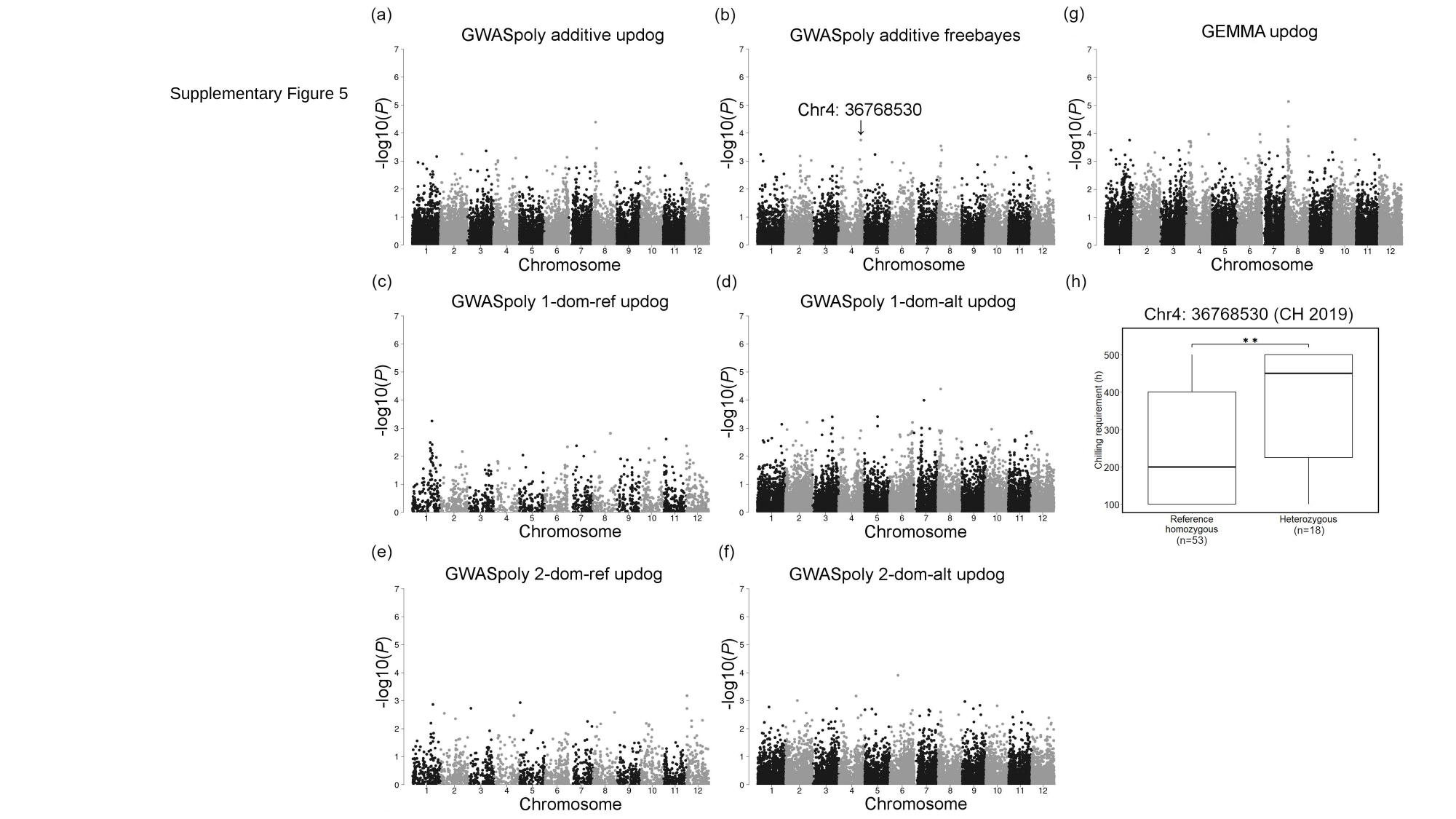

Supplementary Figure 5

## Slide 6
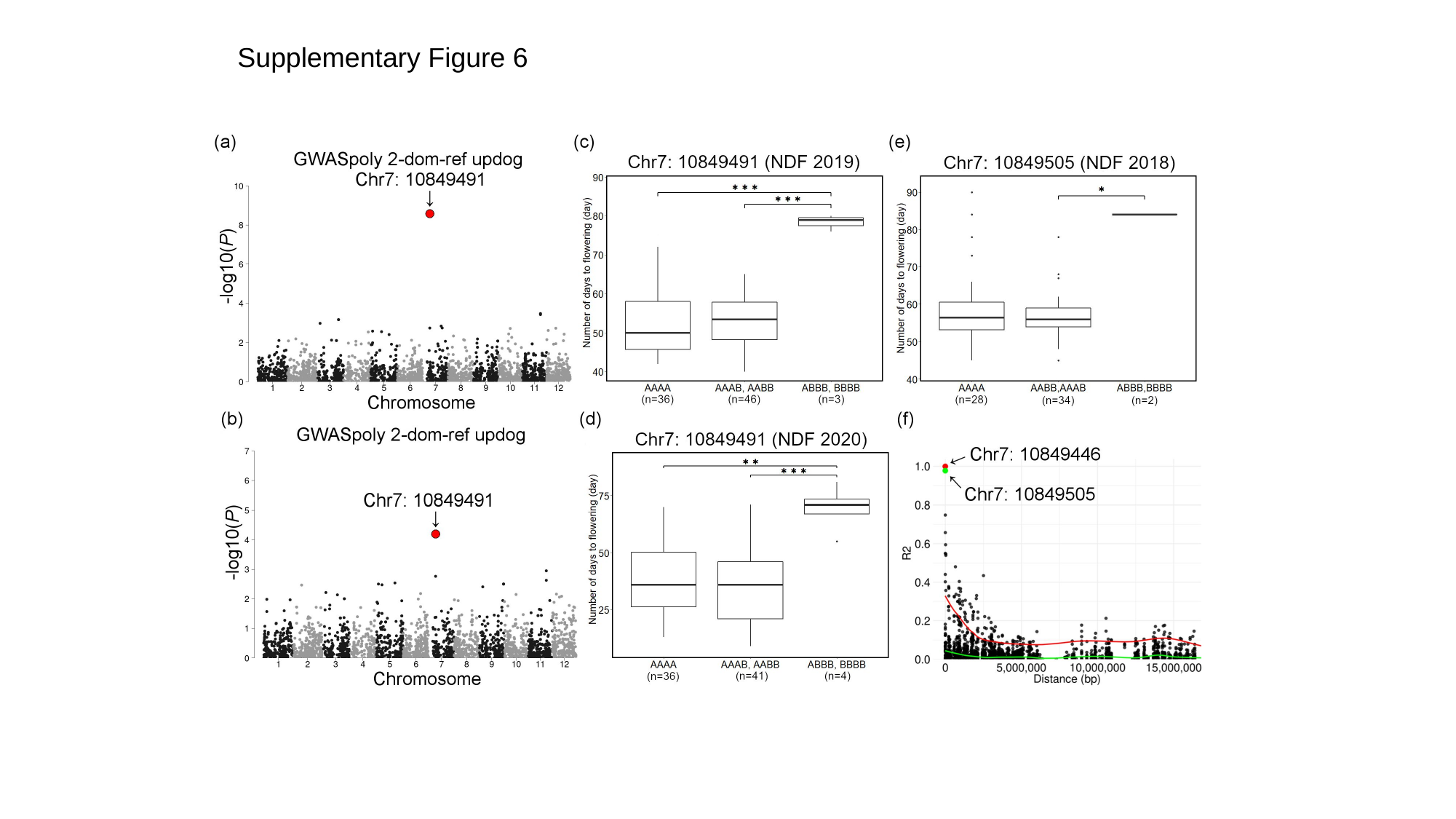

Supplementary Figure 6

## Slide 7
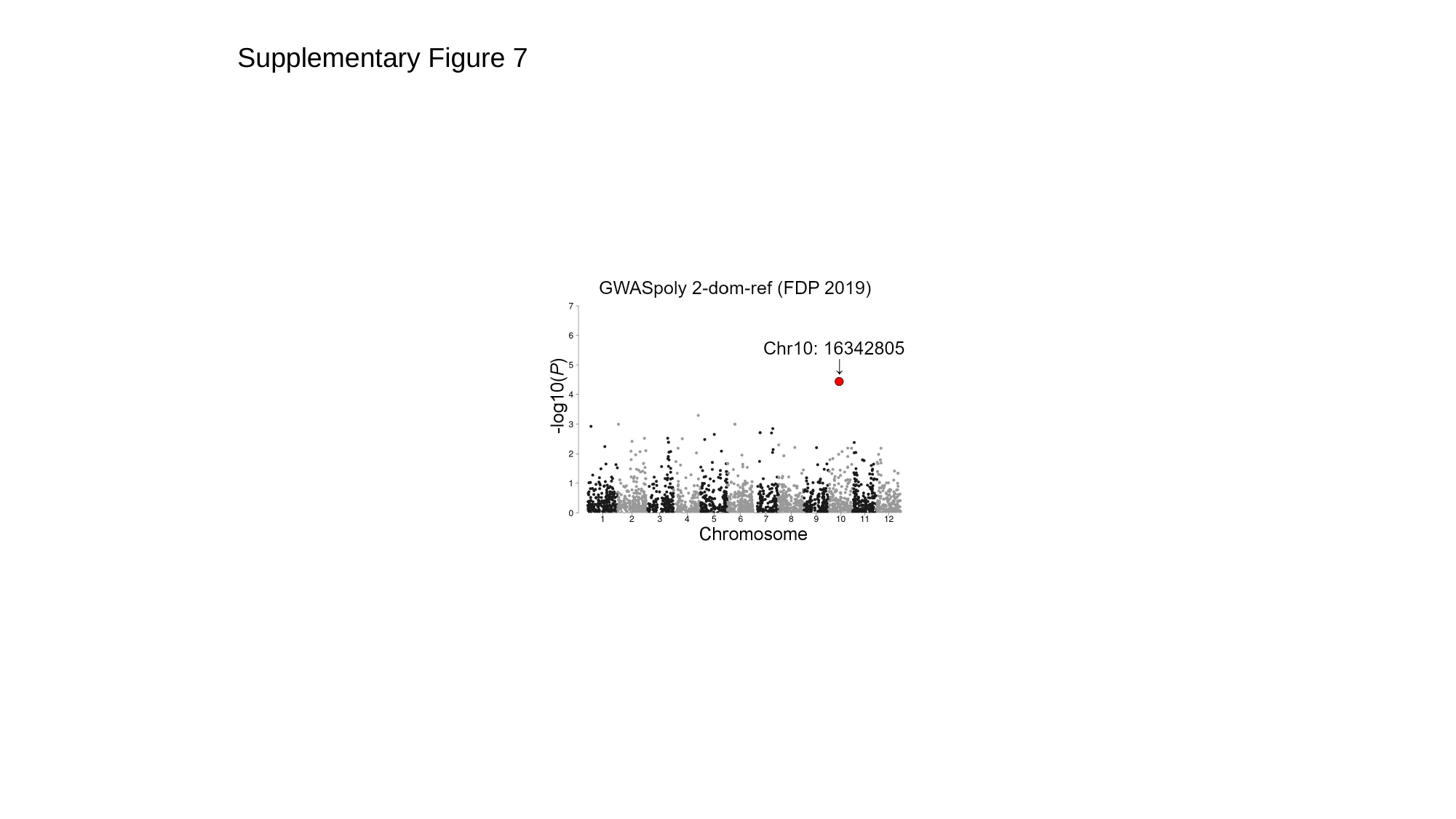

Supplementary Figure 7

## Slide 8
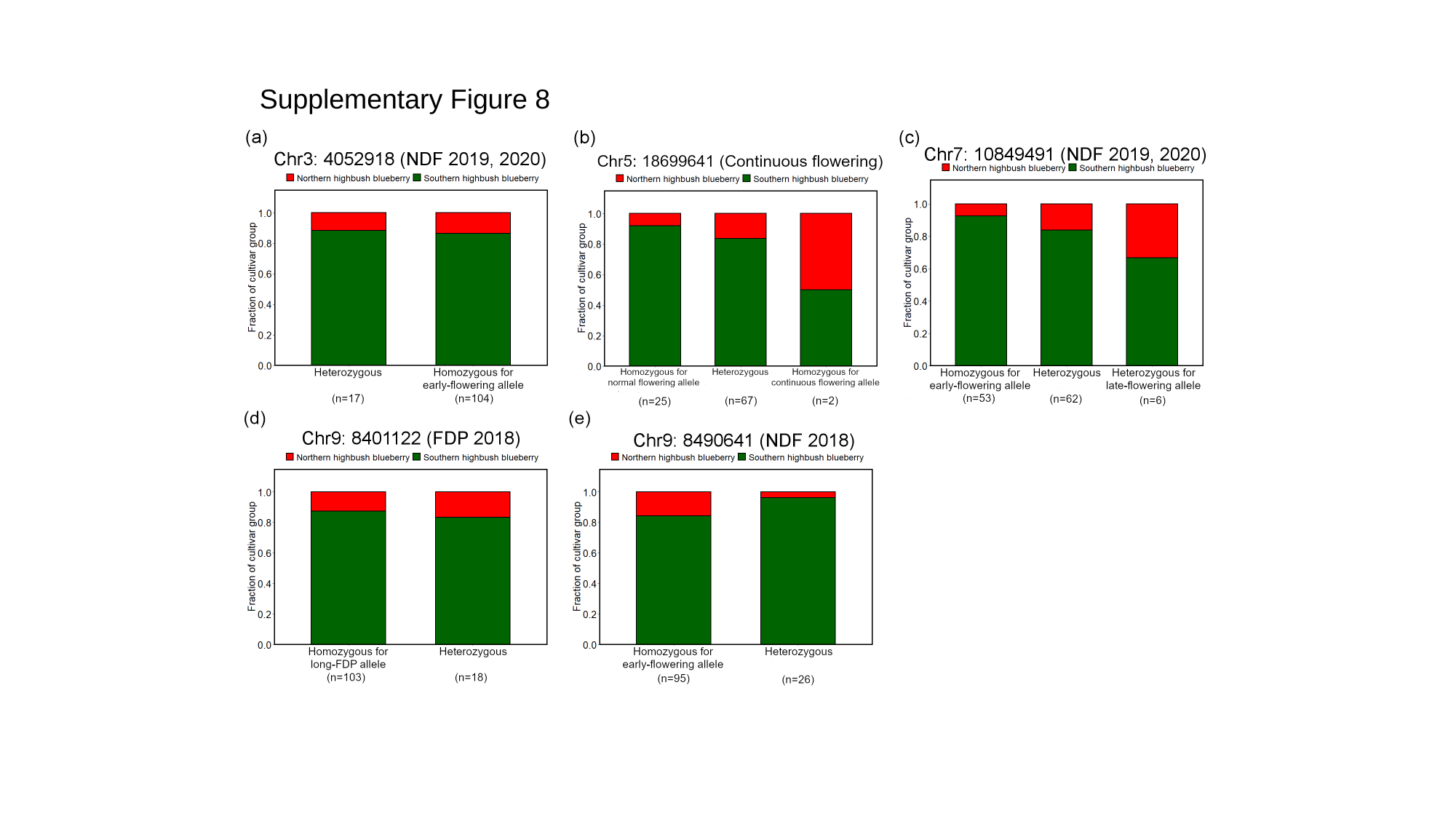

Supplementary Figure 8

## Slide 9
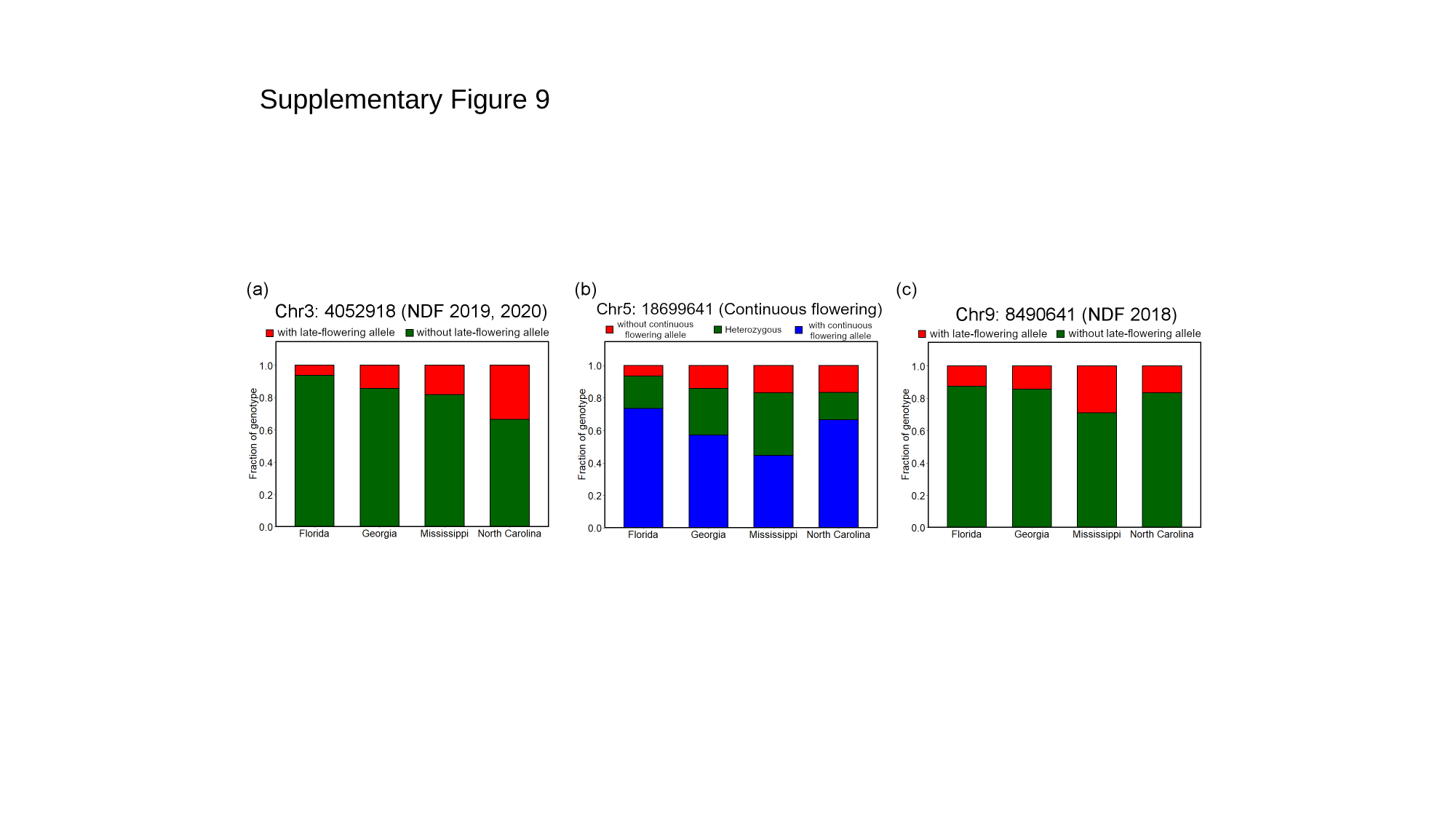

Supplementary Figure 9

## Slide 10
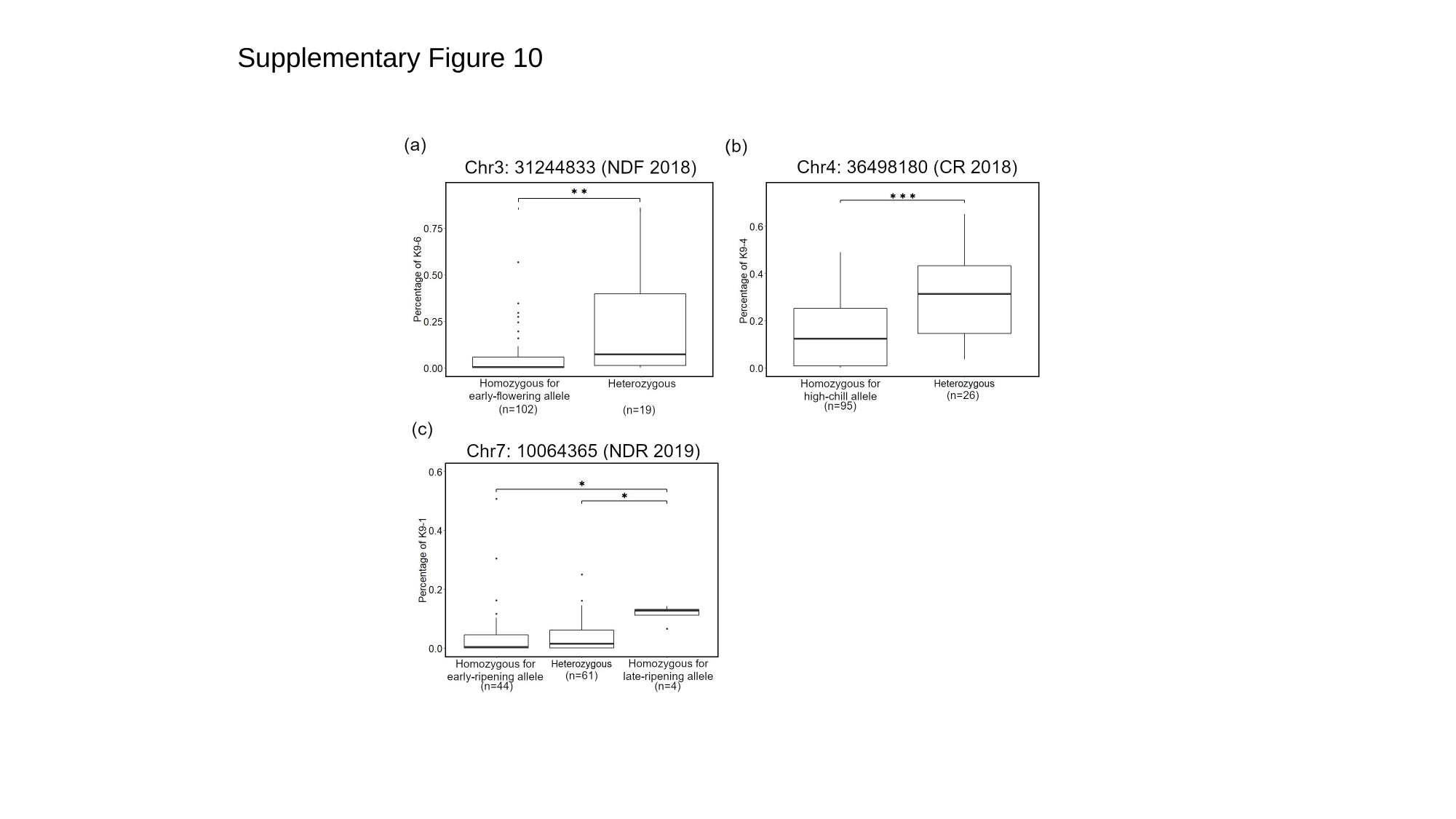

Supplementary Figure 10
